# Supernumerary derivative 22 chromosome resulting from novel constitutional non-Robertsonian translocation: t(20;22)—Case Report

**DOI:** 10.1186/s13039-022-00591-4

**Published:** 2022-03-26

**Authors:** H. C. Manju, Supriya Bevinakoppamath, Deepa Bhat, Akila Prashant, Jayaram S. Kadandale, P. V. V. Gowri Sairam

**Affiliations:** 1 Department of Medical Genetics, JSS Medical College, JSS Academy of Higher Education & Research, Mysuru, India; 2 Department of Anatomy, JSS Medical College, JSS Academy of Higher Education & Research, Mysuru, India; 3 Center for Medical Genetics & Counseling, JSS Hospital, Mysuru, India; 4 Special Interest Group – Human Genomics & Rare Disorders, JSS Academy of Higher Education & Research, Mysuru, India; 5 Department of Biochemistry, JSS Medical College, JSS Academy of Higher Education & Research, Mysuru, India; 6 Center for Human Genetics, Bengaluru, India

**Keywords:** derivative22 chromosome, der(22), sSMC, Supernumerary marker chromosome, t(20;22)

## Abstract

**Background:**

Maternal non-Robertsonian translocation-t(20;22)(q13;q11.2) between chromosomes 20 and 22resulting in an additional complex small supernumerary marker chromosome as derivative (22)inherited to the proband is not been reported yet.

**Case presentation:**

A 4 years old boy with a history of developmental delay, low set ears, and facial dysmorphism was presented to the genetic clinic. Periauricular pit, downward slanting eyes, medially flared eyebrows, downturned mouth corners, and micrognathia were observed. He had congenital heart defect with atrial septal defect (ASD), ventricular septal defect (VSD), and central nervous system (CNS) anomalies with the gross cranium. Karyotype analysis, Fluorescent in-situ hybridization analysis (FISH), and Chromosomal microarray analysis (CMA) were used to determine the chromosomal origin and segmental composition of the derivative 22 chromosome. Karyotype and FISH analyses were performed to confirm the presence of a supernumerary chromosome, and Microarray analysis was performed to rule out copy number variations in the proband's 22q11.2q12 band point. The probands' karyotype revealed the inherited der(22)t(20;22)(q13;q11.2)dmat. Parental karyotype confirmed the mother as the carrier, with balanced non-Robertsonian translocation-46,XX,t(20;22)(q13;q11.2).

**Conclusion:**

The mother had a non-Robertsonian translocation t(20;22)(q13;q11.2) between chromosomes 20 and 22, which resulted in Emanuel syndrome in the proband. The most plausible explanation is 3:1 meiotic malsegregation, which results in the child inheriting derivative chromosome. The parental karyotype study aided in identifying the carrier of the supernumerary der(22), allowing future pregnancies with abnormal offspring to be avoided.

## Background

Small supernumerary marker chromosomes (sSMC) are structurally aberrant chromosomes that are equal in size or smaller than chromosome 20 of the same metaphase spread and cannot be detected or characterized clearly by traditional cytogenetic banding alone. Molecular cytogenetic techniques (including array-based comparative genomic hybridization) are required for their characterization because they are too small to be examined for their chromosomal origin using classic banding techniques [1]. In prenatally determined de novo cases with sSMC, the probability of an aberrant phenotype is estimated to be 13%. This has been refined to 7% for sSMC from chromosome 13, 14, 15, 21, or 22and 28% for all non-acrocentric autosomes [2], with a new suggestion of 30% for all sSMC carriers [3]. Recently, familial sSMC has been discovered to be passed primarily through the maternal line [4]. sSMC is scientifically fascinating since their manner of formation, karyotypic evolution, and the fact that their existence can cause chromosomal imbalances (partial tri-, tetra-, or hexasomies) with no observable clinical effects are still incompletely understood [1, 3, 5]. Investigations in the recent decade have demonstrated the genesis of numerous sSMCs using polymorphic short tandem repeat (STR) markers or SNPs in patients. They've shown that sSMCs can be either maternal or paternal in origin [6–8]. If the origin is maternal, however, it is the product of meiosis I and II errors [9]. Usually, derivative 22 resulting from non-Robertsonian translocation—t(11;22)(q23;q11.2) is observed running in families and gives rise to an extra chromosome in the probands resulting in Emanuel syndrome [10–12]. Palindromic AT-rich regions (PATRR) are found at the breakpoint chromosome 22q11.2, implying that the center of the PATRRs is linked to double-strand breaks (DSBs) that lead to translocation. Various partner chromosomes have also been reported in the previous studies that include chromosomes 3, 4, 6, 8, 9,12, 14,15, 16,17, 19, and X [13–19]. Here we have reported a novel partner chromosome 20, which resulted in der(22) as sSMC, and present as 47,XY, + der(22)t(20;22)(q13;q11.2)dmat.

## Case presentation

**Fig. 1:**
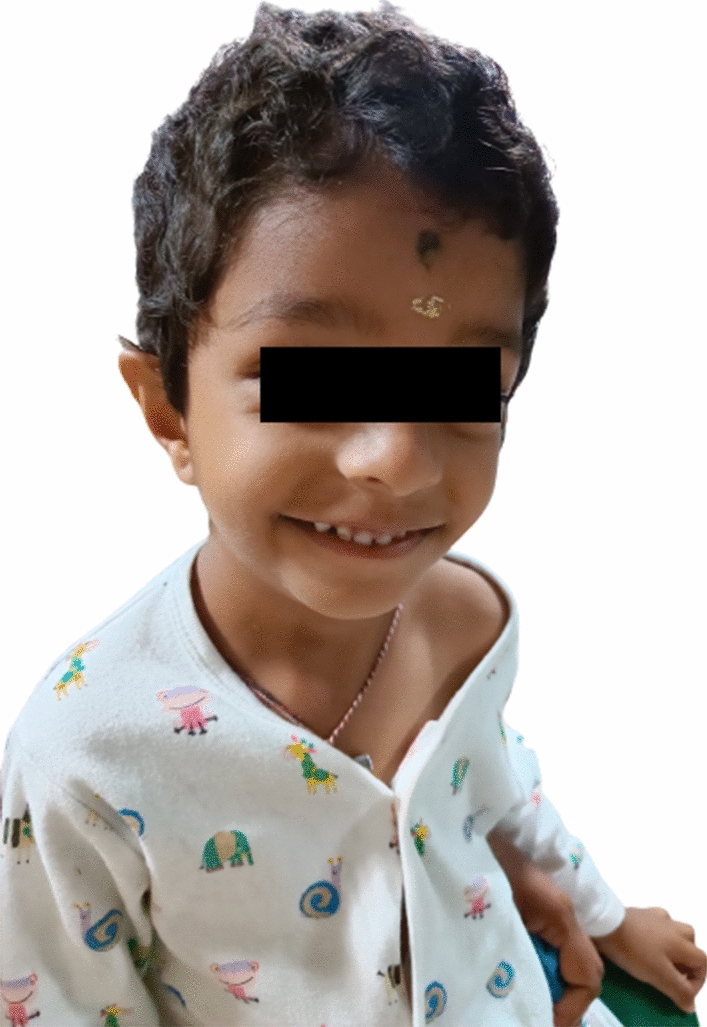
Low set ears

**Fig. 2:**
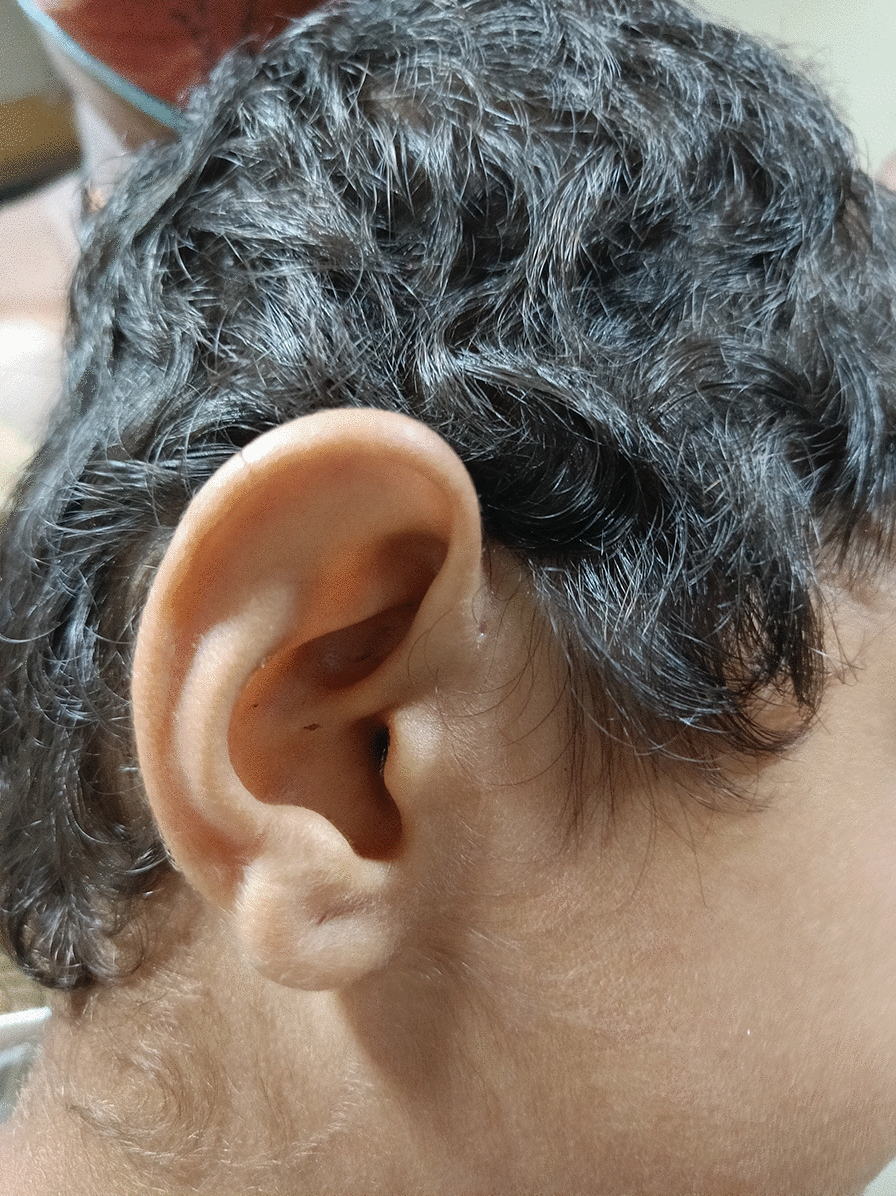
Showing periauricular pit

A 4 years old boy was referred to the Cytogenetics Laboratory at JSS Hospital, Mysore with a history of developmental delay, low set ears, and facial dysmorphism (Fig. 1). He began sitting at the age of two and stood at the age of three years and six months, but he is still unable to stand independently and completely. The child showed a periauricularpit (Fig. 2) and was unable to freely move his right hand. Downward slanting eyes, medially flared eyebrows, downturned mouth corners, and micrognathia were observed. He was the first alive child of the non-consanguineous healthy couple with 30 years old mother, 40 years old father, earlier they had three spontaneous abortions during the first trimester; in the fifth pregnancy, fetal anomalies were discovered at 14.1 weeks, including hypoplastic nasal bone and nuchal thickness of 2.8 and hence the fetus was terminated. The height, weight, and head circumference of the boy were 90 cm (cms), 11.85 kg(kgs), and 47 cm respectively. He had congenital heart defect with atrial septal defect (ASD), ventricular septal defect (VSD), and central nervous system (CNS) anomalies with the gross cranium.MRI findings show supratentorial chronic infarcts in bilateral parieto occipital and superior frontal lobes, as well as volume loss and lateral ventricle dilation in the watershed territory of the brain.

## Methodology

### Cytogenetic analysis

Lymphocytes were cultured from whole blood and stimulated with Phytohemagglutin—M (PHA-M) for 72 h at 37 °C. Colcemid was used to arrest the metaphases in the cultures. The metaphases were isolated using a hypotonic solution(0.075MKCl) and Carnoy's fixative. After GTG banding, chromosomal analysis was performed using Metasystems' Ikaros software.

### *Fluorescence *in situ* hybridization (FISH)*

Fluorescence In Situ Hybridization was performed on cultured pellet from proband to confirm the derivative chromosome 22 using the BCR/ABL1 FISH probes (Metasystems).

### High-resolution chromosomal microarray (CMA)

To ascertain the chromosomal origin and segmental composition of the derivative 22 chromosome, Chromosomal microarray analysis (CMA) was performed using an AffymetrixCytoScan™ 750 K array. The microarray consists of 750 K oligonucleotide probes across the genome, including 550 K unique non-polymorphic probes, and 200 K bi-allelic SNP (single nucleotide polymorphism) probes. Genomic DNA (250 ng) was digested with Nsp1 and ligated using an Nsp1 adapter. Titanium Taq amplified PCR products of size 120 to 2000 bp were purified using AMP pure beads and fragmented to the product size of 25 bp to 125 bp, biotin-labeled, hybridized on CytoScan 750 K gene chip. Data were analyzed using Chromosome Analysis Suite (ChAS) based on the human reference genome (GRCh37/hg 19).

## Results

### Cytogenetic analysis

**Fig. 3:**
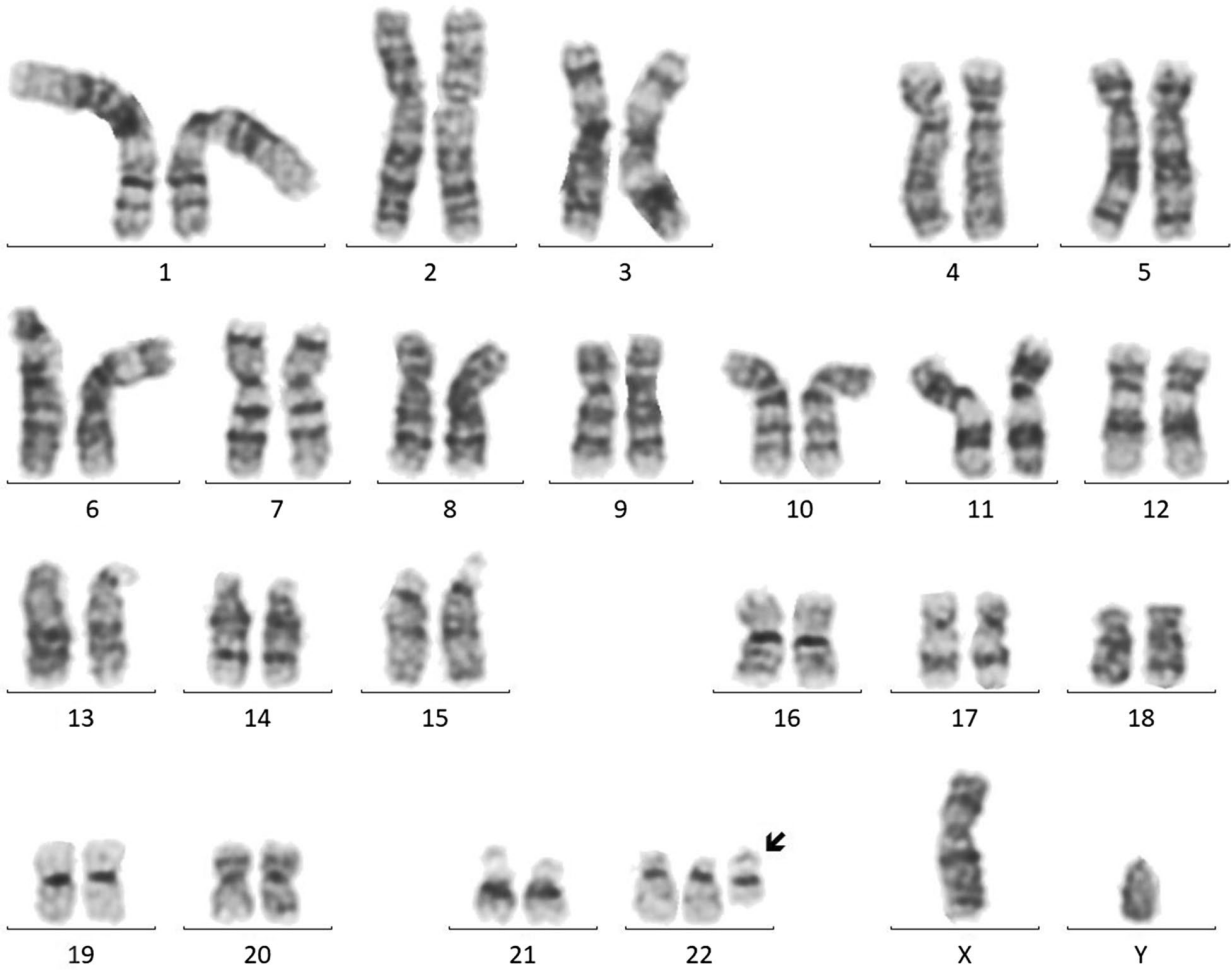
Karyotype of the patient—47,XY, + der(22)t(20;22)(q13;q11.2)dmat

**Fig. 4:**
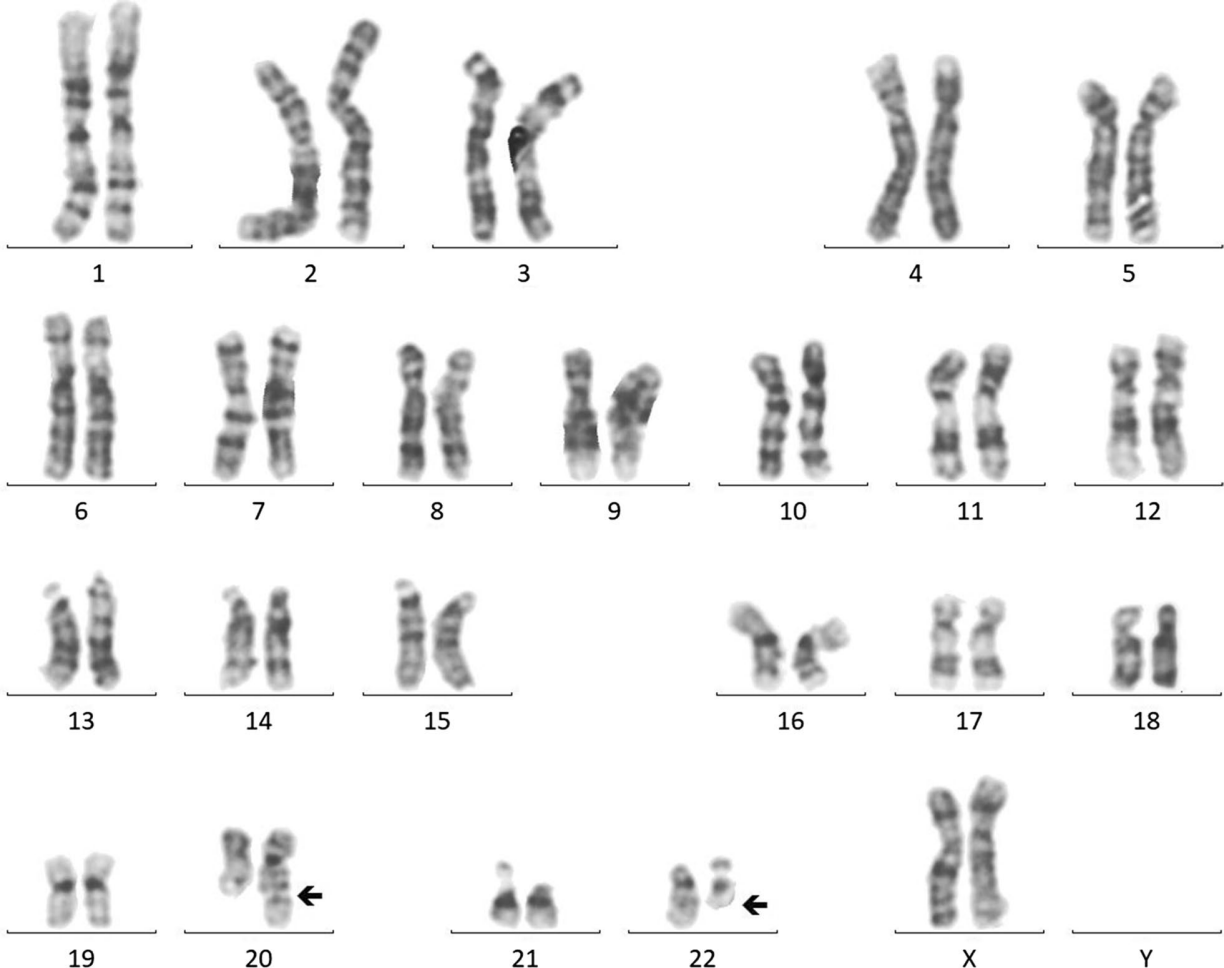
Karyotype of the mother—46,XX,t(20;22)(q13;q11.2)

The GTG banded metaphases were examined. The patient's karyotype was initially reported as abnormal—47,XY, + mar in all analyzed metaphases (Fig. 3). We advocated for parental karyotype to determine the carrier status of the supernumerary marker chromosome. The mother showed an abnormal female karyotype with a balanced translocation between the long arm of chromosome 20 at q13 and the long arm of chromosome 22 at q11.2—46,XX,t(20;22)(q13;q11.2) (Fig. 4), whereas, father’s karyotype was normal confirming that the mother was a carrier and transmitted the supernumerary derivative 22 chromosome to the child. Thereafter, the karyotype of the child was considered to be 47,XY, + der(22)t(20;22)(q13;q11.2)dmat.

### *Fluorescence *in situ* hybridization (FISH)*

**Fig. 5:**
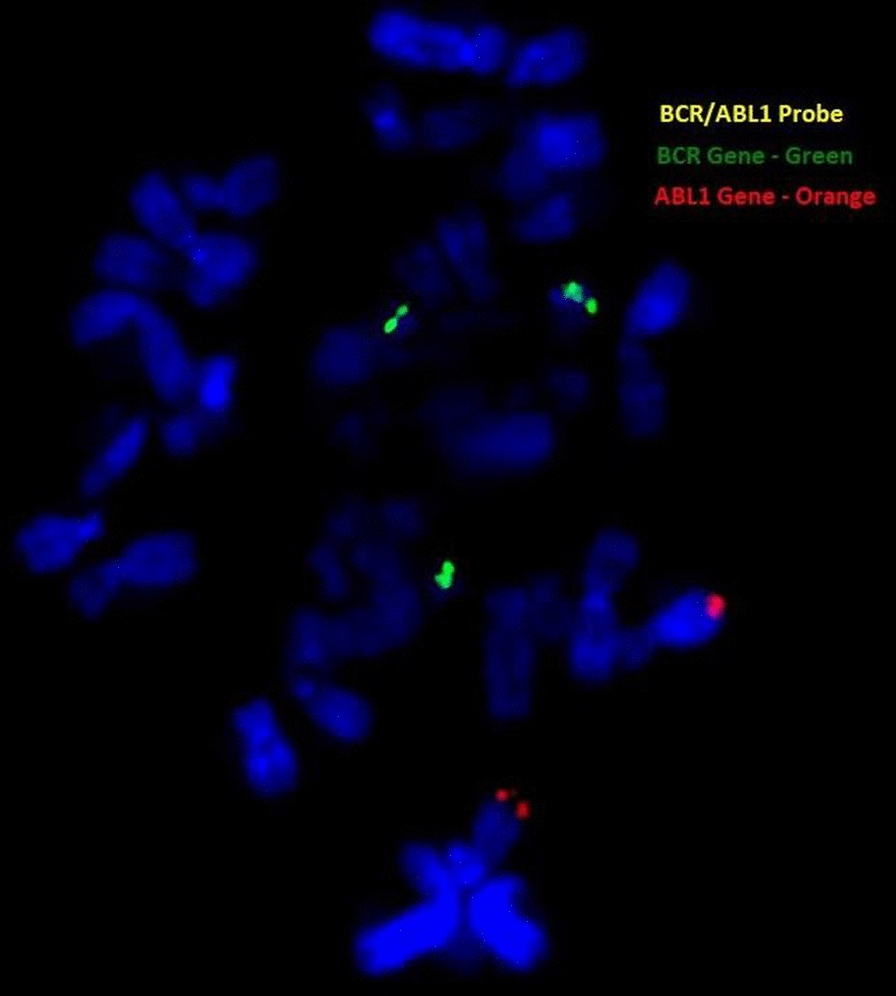
FISH showing 3 green signals (BCR gene on 22q11.2)

We used a BCR/ABL1 dual fusion probe with an orange probe hybridizing to the ABL1 gene region at 9q34.1 and a green probe hybridizing to the BCR gene region at 22q11.2. Metaphase revealed three green signals indicating that sSMC is corresponding to chromosome 22q11.2 (Fig. 5).

### Chromosomal microarray (CMA)

**Fig. 6:**
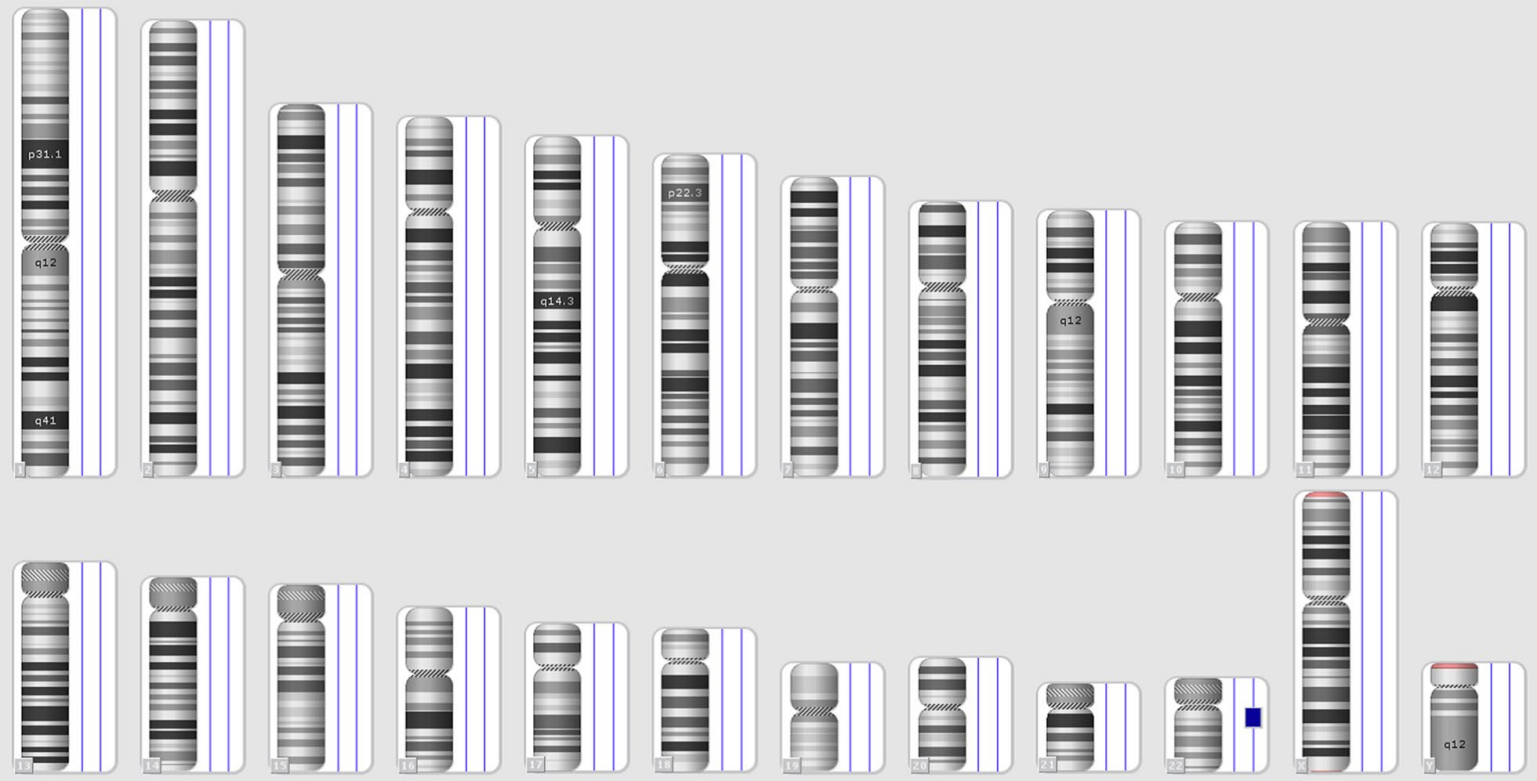
Karyoview

**Fig. 7:**
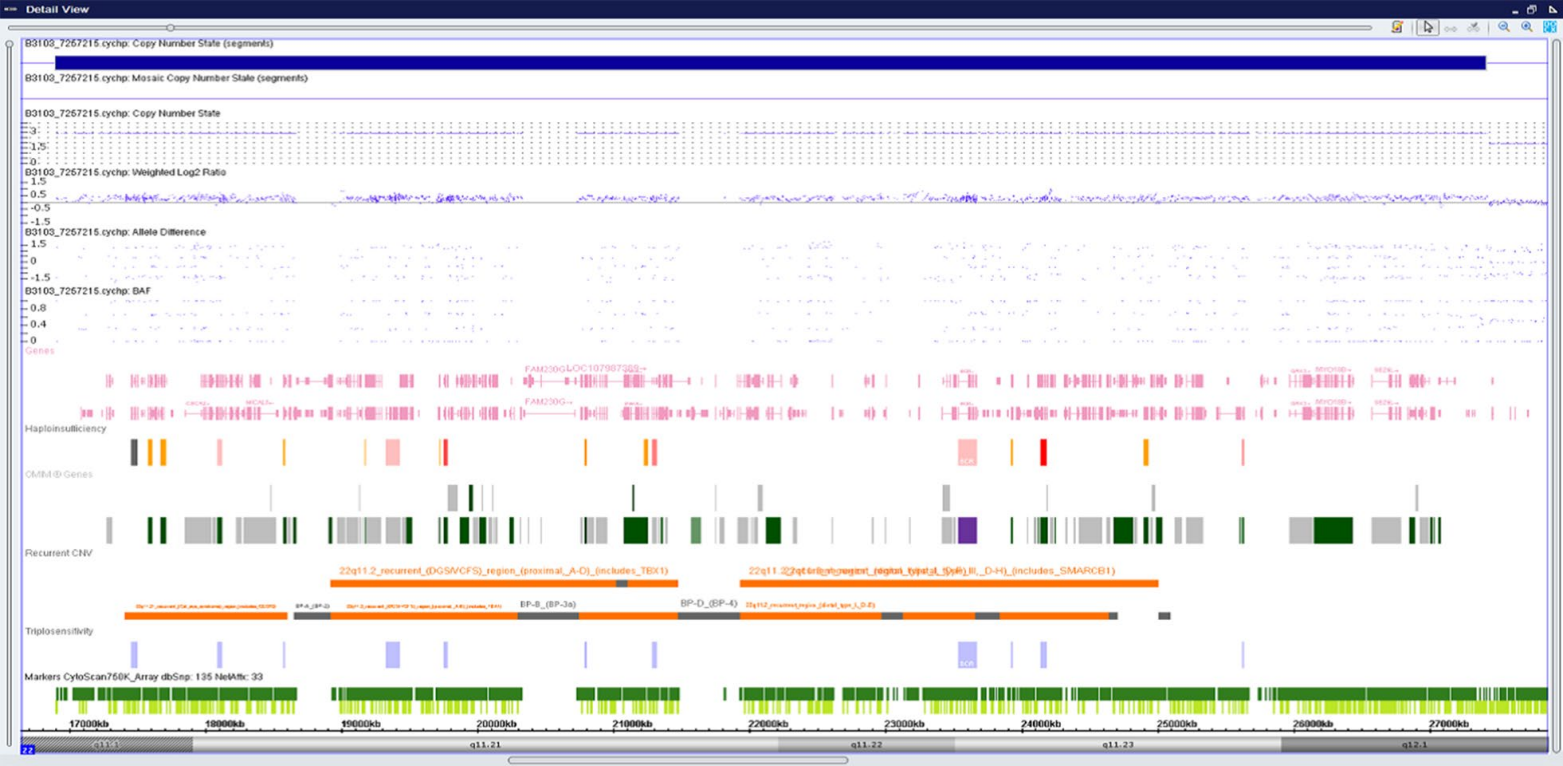
Detailed view_22q11.2q12.1gain

The cytogenomic microarray analysis revealed a 10.5 Mb gain involving chromosome 22 within cytoregion 22q11.2q12.1, indicating trisomy of this region (Fig. 6 and 7). There are 110 OMIM genes in this region. Developmental delay, intellectual disability/learning disability, and congenital heart anomaly are all symptoms of chromosome 22q11.2q12.1duplication syndrome. The majority of the genes are linked to the DiGeorge Syndrome/Velocardiofacial Syndrome genes.

### KaryoviewCNVs6

See Figs. 6 and 7.

## Discussion

Approximately 9% of all sSMCs arise from chromosome 22 [17].The supernumerary-der(22) syndrome has been thought to be due to the error in the meiosis. The widely known recurrent non-Robertsonian translocation in humans is the result of 3:1 meiotic malsegregation in carriers of the constitutional t(11;22)(q23;q11.2) translocation [20].In around 99% of cases, the extra derivative 22 chromosome rises during 3:1 meiotic malsegregation in one of the parents [11]. Usually, supernumerary derivative 22 chromosome is resulting in parents who had t(11;22)(q23;q11.2). This chromosomal rearrangement is a very common non-Robertsonian translocation and the carrier of the proband's derivative 22 chromosome.

Female carriers of the t(11;22) have a nearly 4% chance of having children with the supernumerary derivative chromosome 22 syndrome, while male carriers have a 0.7 percent chance [21]. The palindromic AT-rich regions (PATRR) have been located at both breakpoints of chromosome 11q23 and chromosome 22q11.2 [14, 16, 18]. Chromosome 22 at q11.2 breakpoint is involved in translocations with other partner chromosomes 4, 6, 8, 9, 12, 14, 15, 16, 17,19 and X with translocations at t(4;22)(p15.2;q11.2),t(6;22)(p22.1;q11.2), t(8;22)(q24.13;q11.2),t(8;22)(p22;q11.21), t(9;22)(p13;q11.2), t(12;22)(p12-p13.3;q11.2), t(14;22)(q31;q11.2), t(15;22)(q26.1;q11.21), t(16;22)(p13.3;q11.21), t(17;22)(q11;q11.2), t(19;22)(q13.42;q11.2) and t(X;22)(p22.3;q11.2) respectively [13, 14, 16, 17, 23–31]. In addition, another chromosomal rearrangement t(3;22)(q28;q13.3) is resulting into sSMC other than break point 22q11.2 [15]. Previously reported case shows sSMC resulting due to paternal uniparental disomy of chromosome 22 [32]. Present finding t(20;22)(q13;q11.2) is the new partner chromosome mediating to form supernumerary derivative chromosome 22 in offspring. Several clinical features of the patient overlap with those of the Emanuel syndrome. Overdosage of genes on the derivative 22 chromosome affects clinical features such as facial dysmorphism, developmental delay, micrognathia, congenital heart defect, and CNS malformation.

## Conclusion

The clinical aspects of derivative 22 chromosome syndrome overlap with those of Emanuel syndrome, regardless of carrier chromosomal rearrangement. Most of the supernumerary der(22) chromosome is forming due to any one of the parents being the carrier. A non-Robertsonian translocation involving 22q11.2.A translocation had occurred in the mother between chromosomes 20 and 22 in our case. The parental karyotype analysis was effective in identifying the carrier of the supernumerary der(22).To minimize future pregnancies with defective progeny, prenatal karyotyping is essential.

## Data Availability

Not applicable to this article as no datasets were generated or analyzed during the current study.

## Abbreviations

ABL1: V-Abl Abelson murine leukemia viral oncogene homolog-1
ASD: Atrial septal defect
BCR: Breakpoint cluster region
bp: Basepair
ChAS: Chromosome analysis suite
CMA: High-resolution chromosomal microarray
cms: Centimeters
CNS: Central nervous system
der: Derivative
dmat: Derivied from chromosomal abnormality of maternal origin
DNA: Deoxy ribonucleic acid
DSB: Double-strand breaks
FISH: Fluorescence In Situ Hybridization
GTG: G bands with trypsin and giemsa stain
KCl: Potassium chloride
Kgs: Kilograms
mar: Marker
ng: Nanogram
OMIM: Online mendelian inheritance in man
PATRR: Palindromic AT-rich regions
PCR: Polymerse chain reaction
PHA-M: Phytohemagglutin M form
SNP: Single nucleotide polymorphism
sSMC: Small supernumerary marker chromosome
VSD: Ventricular septal defect
